# Clinical Characteristics of Wolfram Syndrome in Chinese Population and a Novel Frameshift Mutation in *WFS1*

**DOI:** 10.3389/fendo.2018.00018

**Published:** 2018-02-12

**Authors:** Lian Duan, Qian Li, An-Li Tong, Jiang-Feng Mao, Miao Yu, Tao Yuan, Xiao-Feng Chai, Feng Gu

**Affiliations:** ^1^Key Laboratory of Endocrinology, Ministry of Health, Department of Endocrinology, Peking Union Medical College Hospital, Peking Union Medical College and Chinese Academy of Medical Sciences, Beijing, China

**Keywords:** Wolfram syndrome, DIDMOAD, *WFS1*, diabetes mellitus, optic atrophy

## Abstract

**Objective:**

Wolfram syndrome (WS) is a rare, degenerative, and hereditary disorder characterized by ear diabetes mellitus (DM) and optic atrophy (OA). We aim to characterize clinical features in Chinese patients who had been poorly studied until now.

**Methods:**

We performed a retrospective review of patients with WS seen in the Peking Union Medical College Hospital from 2002 to 2017. Data including demographic data, clinical presentations, examination results, family history, and genetic analysis were described.

**Results:**

Six patients with WS were identified, meeting the diagnostic criteria of the coincidence of DM and OA before 15 years old or the existence of two *WFS1* mutations. All were male, with the median age of 14.5 years (range 10–19 years). Blood glucose impairment, OA, and diabetes insipidus were present in all (100%), hearing impairment in four (66.7%), urological abnormalities in four (66.7%), neurological abnormalities in one (16.7%), and endocrine disorder in one (16.7%). Rare presentation includes cataract, glaucoma, and spina bifida occulta. Diabetes was insulin-dependent and not ketosis onset, with antibody to glutamic acid decarboxylase and islet cell negative. Genetic analysis revealed mutations in *WFS1* in three patients. A novel frameshift mutation (p.Asp151Glufs*93) was identified in exon 4 of *WFS1*.

**Conclusion:**

Our series of WS patients indicated that WS is a degenerative disease with a wide and variable spectrum, characterized by ear non-autoimmune DM and bilateral OA. Genetic analysis is recommended when suspected of WS.

## Introduction

Wolfram syndrome (WS) is a rare, degenerative, and hereditary disease described by Wolfram and Wagener in 1938 for the first time. Its prevalence was estimated to be 1 in 68,000 to 1 in 770,000 (1, 2). WS has a wide spectrum of manifestations, known as DIDMOAD [diabetes insipidus (DI), diabetes mellitus (DM), optic atrophy (OA) and deafness], also including urological abnormalities, psychological abnormalities, and endocrine disorders. The minimum diagnostic criteria of WS are the coincidence of early-onset DM and OA (2). There is no effective treatment to prevent disease from progression with aging, reporting a median life expectancy of 30 years (range 25–49) (2).

*WFS1* was identified as the first causative gene of WS in 1998, mutated in almost all patients (3, 4). *CISD2* was described as the second responsible gene in some Jordanian families in 2007 (5, 6). *WFS1* encodes Wolframin, an endoplasmic reticulum (ER) transmembrane protein, generally expressed in a variety of tissues, highly in pancreatic β-cells and brain (4). Its function has not been fully clarified, including a crucial role in the negative regulation of ER stress (7), the regulation of insulin biosynthesis and secretion in pancreatic β-cells (8, 9), the regulation of Ca^2+^ homeostasis (10), and the regulation of Na^+^/K^+^ ATPase β-1 subunit (11). *CISD2* encodes Endoplasmic Reticulum Intermembrane Small protein, involved in mitochondrial disorder, associated with a variant feature of gastrointestinal ulcer and bleeding tendency without DI (12, 13). Although loss-of-function mutation in *WFS1* is related to autosome-recessive WS, a mutation in *WFS1* was also believed to be a cause of autosome-dominant OA, hearing impairment, and diabetes (14, 15). To date, approximately 350 mutations in *WFS1* and 3 mutations in *CISD2* are reported in HGMD database. Previous studies implicate that the classification of *WFS1* mutations may predict the onset age of symptoms (16). The underlying mechanisms of WS as well as associations between genotype and phenotype remain to be further elucidated.

The natural history of WS has been characterized in different studies, with the maximum series including 67 patients (17). Very limited data were concerning WS Chinese patients, almost a few single cases. We describe the clinical features of a series of patients in the last 15 years at a tertiary clinic center in China.

## Patients and Methods

To retrospectively describe all WS patients evaluated in the Peking Union Medical College Hospital (PUMCH), Beijing, between June 2002 and 2017, the electronic medical record query system was searched for “Wolfram syndrome” or “DIDMOAD syndrome” after approval from the Institutional Review Board and Ethics Committee of PUMCH. Patients were eligible for our study if the diagnostic criteria of “the coincidence of DM and OA before 15 years old or identification of 2 *WFS1* mutations” are fulfilled after they or their parents were consent to participate.

Medical records were reviewed to extract information, consisting of demographic data, clinical presentation, including symptoms, organ system involved, the corresponding onset age and examination results, family history, and genetic analysis.

Proteinuria was defined biochemically as urine albumin-to-creatinine ratio is greater than 30 mg/g or 24-h protein quantity is greater than 150 mg. Fundus was evaluated for features suggestive of OA as well as diabetic retinopathy by ophthalmologists. Hearing assessment is based on pure tone test and acoustic immittance measurement. Water deprivation tests followed by pituitrin or minirin administration were performed in all patients. Central DI was diagnosed when urine osmotic pressure was elevated and urine volume was reduced after the administration of pituitrin or minirin. GH deficiency was diagnosed on the basis of abnormally low IGF-1 compared to the referenced value of the same age and gender group. Adrenal dysfunction was diagnosed when a morning cortisol was lower than 5 g/dl, low or inappropriate normal ACTH prompting secondary insufficiency. Hypothyroidism was evaluated on the basis of FT3, FT4, and TSH. Hypogonadism in male was diagnosed when serum total testosterone was lower than 300 ng/dl. Serum luteinizing hormone and follicle-stimulating hormone were measured to distinguish primary hypogonadism from secondary. Urinary tract abnormality was considered when ultrasonography or computed tomography (CT) indicates urinary tract dilatation or increased residual urine volume.

Pituitary magnetic resonance imaging (MRI) was performed in all patients. Brain MRI was performed in patients suspected of neurological symptoms, and psychological evaluation by psychologists was performed in patients suspected of psychological symptoms. Genetic analysis was performed as the following: The peripheral blood DNA was extracted, and primers were designed targeting exons and the exon–intron sequences of WFS1. Then, the genes were amplified by PCR, the products were sent for Sanger sequencing, and the results were analyzed. Results available were reviewed. Telephone follow-up was made to evaluate the survival.

Descriptive statistics were applied to characterize results. If age was provided for more than one presentation that fit into the same clinical feature, then the minimum age was taken as the onset age.

## Results

### Patient Features and Clinical Presentations

Seven patients with WS were retrieved through the search system. One patient clinically suspected of WS, presenting polydipsia, polyuria, and hearing impairment, was excluded because of lack of co-occurrences of DM and OA as well as *WFS1* mutation. Detailed medical records of six patients were reviewed and described.

The demographic features of the patients and detailed clinical presentations are shown in Table 1. All patients were male. The median ages of patients were 14.5 years (range 10–19 years). All the six patients had four to six clinical features. Blood glucose impairment, OA, and DI were present in all patients, one of them had impaired glucose tolerance (IGT) rather than DM. Four of the six patients (66.7%) had hearing impairment, also had the full DIDMOAD phenotype. Urological abnormalities were present in four of the six patients (66.7%). One patient (Case 4) also had hypergonadotropic hypogonadism suggestive of gonado affected, and hydrocephalus, inarticulate speech, and cognitive disorders suggestive of nerve system affected. More rare presentations including cataract, glaucoma, and spina bifida occulta were found in one patient, respectively.

**Table 1:** Demographic and clinical characteristics in patients with WS.

Case no.	Age, years	Sex	DM/IGT	OA	DI	HL	UD	H	ND	PD	Other features
1	10	M	Yes	Yes	Yes	Yes	No	No	No	No	None
2	15	M	Yes	Yes	Yes	Yes	Yes	No	No	No	Cataract
3	10	M	Yes	Yes	Yes	Yes	Yes	No	No	No	None
4	19	M	Yes	Yes	Yes	No	No	Yes	Yes	No	None
5	15	M	Yes	Yes	Yes	No	Yes	No	No	No	Glaucoma
6	14	M	Yes	Yes	Yes	Yes	Yes	No	No	No	Spina bifida occulta

*M, male; DM, diabetes mellitus; IGT, impaired glucose tolerance; OA, optic atrophy; DI, diabetes insipidus; HL, hearing loss; UD, urological disorder; H, hypogonadism; ND, neurological disorder; PD, psychological disorder*.

### Onset-Age Distribution of Clinical Presentation

**Figure 1:**
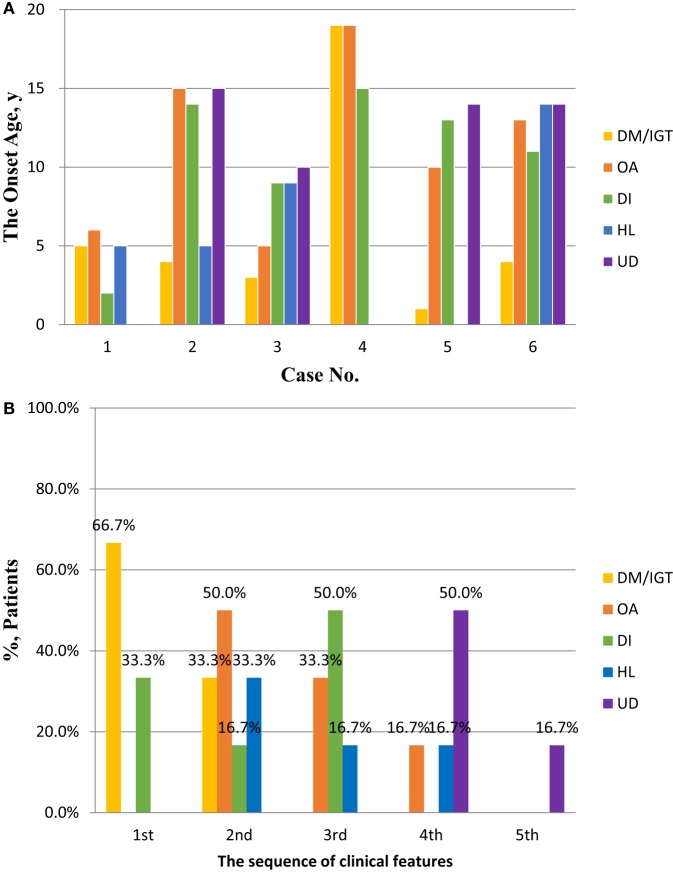
The onset ages and sequence of clinical features in patients with Wolfram syndrome (WS). **(A)** The onset ages of DM/IGT, OA, DI, HL, and UD in patients with WS. Detailed descriptive statistics are shown in Table S1 in Supplementary Material. **(B)** The sequence of DM/IGT, OA, DI, HL, and UD in patients with WS. Abbreviations: DM, diabetes mellitus; IGT, impaired glucose tolerance; OA, optic atrophy; DI, diabetes insipidus; HL, hearing loss; UD, urological disorder.

Diabetes mellitus was developed at the median age of 4 years (range 1–5 years) in five of the six patients, and the remaining one developed IGT in 19 years. Hearing loss (HL) was developed at the median age of 7.7 years (range 5–14 years). The median age of OA, DI, and urological disorders (UDs) at onset were 11.5 (range 5–19 years), 12 (range 2–15 years), and 14 (range 10–15 years) years, respectively. DM or IGT was present as the first or second clinical features. The onset-age distribution of clinical futures in WS is shown in Figure 1. Detailed age statistics are provided in Table S1 in Supplementary Material.

### Characteristics of DM

The five patients with diabetes were not ketosis onset, with antibody to glutamic acid decarboxylase and islet cell all negative. C-peptide was lower (range 0–0.13 ng/ml) and all were insulin-dependent. The median HbA1c was 8% (range 7–11.1%). All patients were treated by insulin. The occurrence of hypoglycemia was reported in all individuals. Regarding diabetic microangiopathic complications, moderately increased albuminuria was found in Case 5 that had diabetes for 14 years, and nonproliferative retinopathy was found in Case 6 that had diabetes for 10 years. All are shown in Table 2.

**Table 2:** Clinical features of DM in patients with Wolfram syndrome.

Case no.	Duration, years	Ketosis at onset	C-P	HbA1c, %	GADA/ICA	Hypoglycemia	Retinopathy/nephropathy	DM family history
1	5	No	0.07	8.0	NEG	Yes	No	Yes
2	6	No	0.06	7.5	NEG	Yes	No	Yes
3	7	No	0.13	8.6	NEG	Yes	No	No
5	14	No	–	7.0	NEG	Yes	Moderately increased albuminuria	Yes
6	10	No	0.00	11.1	NEG	Yes	Nonproliferative retinopathy	No

*C-P, C-peptide; GADA, glutamic acid decarboxylase antibody; ICA, islet cell antibody; NEG, negative; DM, diabetes mellitus*.

### Other Clinical Features

Optic atrophy was bilateral and progressive in all six patients, could present as hypopsia, visual field defect, and color blindness. High-frequency sensorineural HL was found at the beginning in the four patients, and low—frequency hearing was also found damaged with progressing in Case 1.

**Figure 2:**
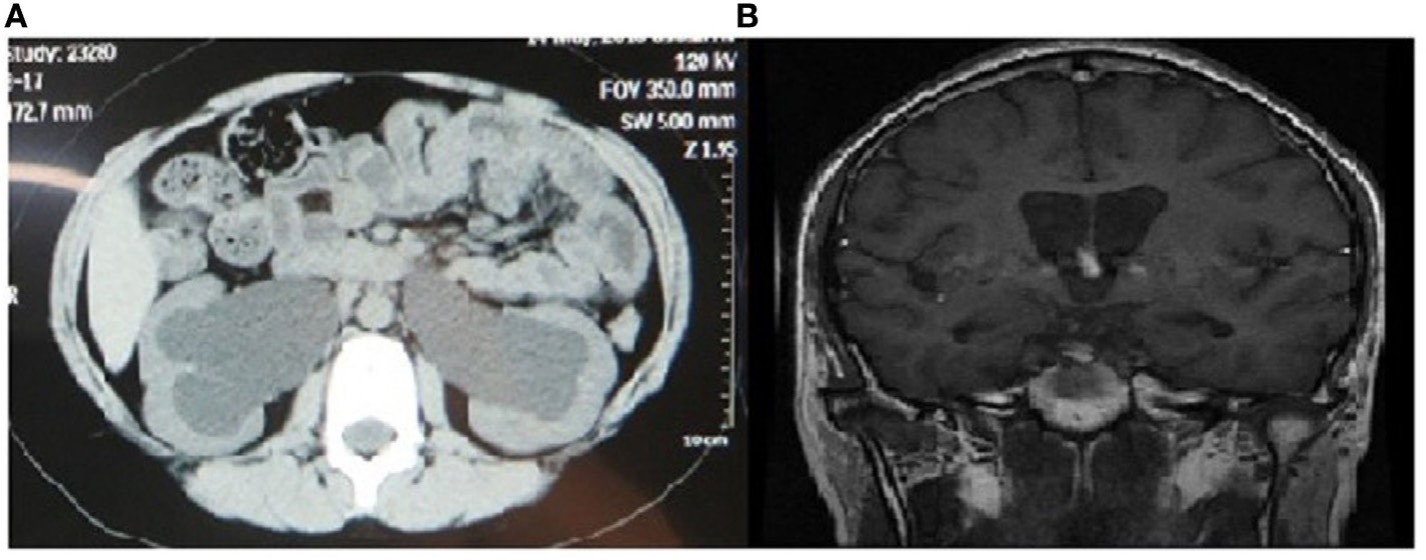
Image findings of urinary and nerve systems in patients with Wolfram syndrome. **(A)** Abdominal computed tomography (plain scan view) showing bilateral dilated renal pelvis of Case 3 in 10 years. **(B)** Brain magnetic resonance imaging (coronal/sagittal view) showing cerebral ventricle dilation, bulging corpus callosum, and hydrocephalus.

Urological abnormalities including dilation of urinary tract and residual urine in bladder were established by imaging examinations in four of the six patients. As shown in Figure 2A, hydronephrosis was found in the CT image of Case 3 in his 10 years, with a residual urine volume of 305 ml in the bladder according to ultrasound results. The simultaneous adjusted glomerular filtration rate for children was 52.73 ml/min/1.73 m^2^, which normalized after urine catheterization, implicating acute renal dysfunction and indication of cystostomy.

Inarticulate speech and cognitive disorders were found in Case 4, with cerebral ventricle dilation, bulging corpus callosum, and hydrocephalus presentations in MRI image. The posterior pituitary signal also disappeared on T1-weighted MRI image. MRI image is shown in Figure 2B.

The overall survival rate was 100% at the time of follow-up in July 2017, and the age of patients ranging from 11 to 24 years.

### Genetic Analysis

**Figure 3:**
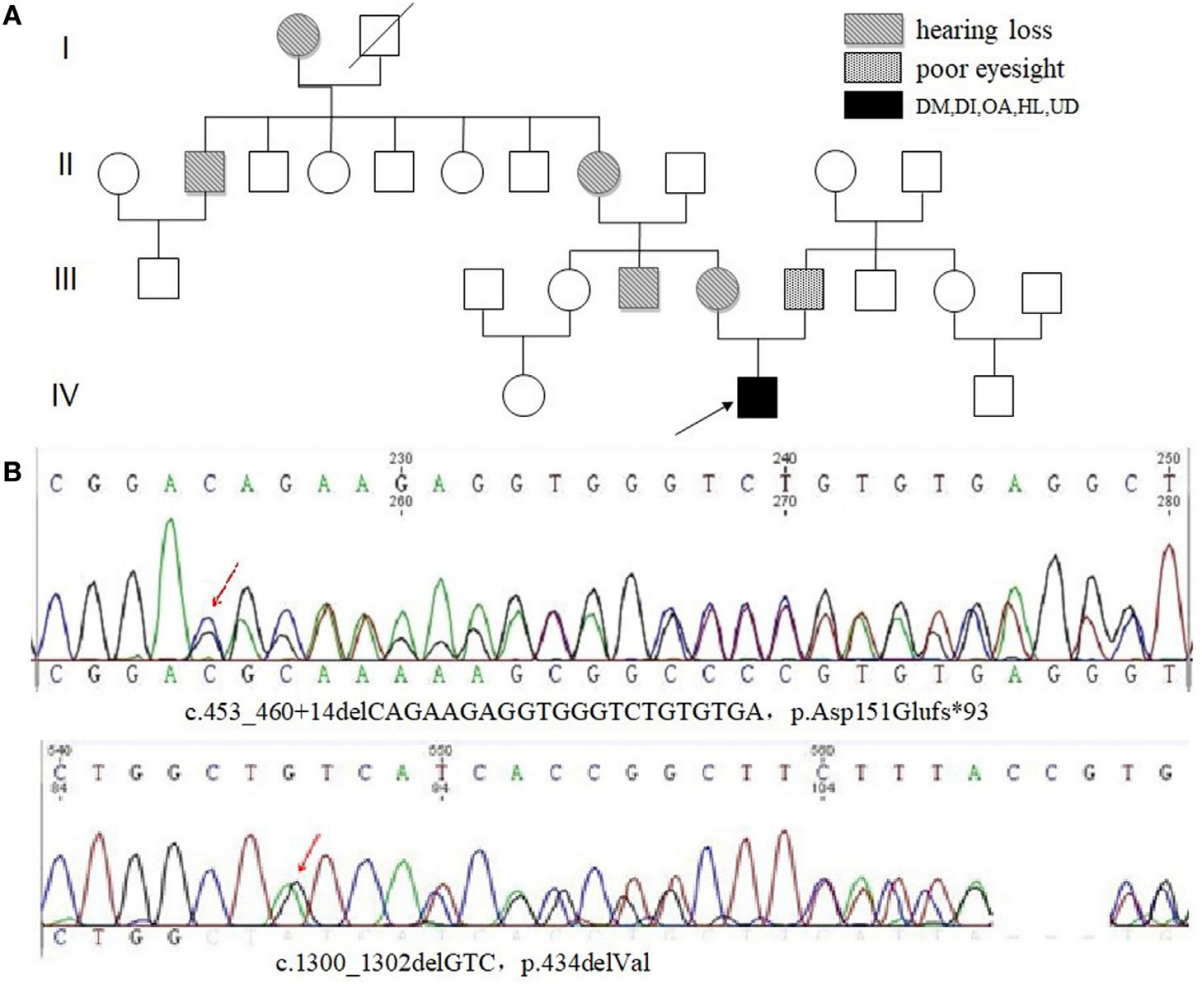
Family pedigree of Case 3 with Wolfram syndrome and the results of genetic analysis. **(A)** The proband (Subject IV-2) was indicated by a black arrow. HL or poor eyesight was present in some family members. **(B)** Sanger sequencing of genomic *WFS1* DNA showed compound heterozygous mutations, p.Asp151Glufs*93 and p.434delVal in proband (Subject IV-2). Abbreviations: DM, diabetes mellitus; IGT, impaired glucose tolerance; OA, optic atrophy; DI, diabetes insipidus; HL, hearing loss; UD, urological disorder.

*WFS1* gene mutation in peripheral blood DNA was detected in four of six patients (67%). There was no pathogenic mutation detected in Case 1, with no further genetic analysis. Two were identified harboring three pathogenic mutations in exon 8, with detailed results unavailable. The results of Case 3 are shown in Figures 3A,B. Compound heterozygotic mutations were identified and inherited from his father and mother, respectively. The father (Subject III-6) of the proband had a complaint of poor eyesight in childhood. The mother (Subject III-5) of the proband had a complaint of HL, as well as his mother’s brother (Subject III-4), grandmother (Subject II-8), and other relatives (Subject I-1 and II-2). Results of mutation detection are shown in Figure 3B, including one deletion mutation (p.434delVal) in exon 8 and one novel frameshift mutation in exon 4 caused by the deletion of 21 nucleotides deletion (p.Asp151Glufs*93). The former mutation was also identified in his father (Subject III-6). The latter was identified in his mother (Subject III-5). There was no mutation detected in his aunt (Subject III-3) who had no HL.

## Discussion

Wolfram syndrome is a rare, autosome-recessive hereditary disease. In this study, we retrospectively described the series of six WS patients from a single center in the last 15 years from China, characterized clinical features, and reported a novel *WFS*1 mutation.

Diabetes mellitus was the most common clinical feature in our cohort, resonating with previous studies. Similarly, OA, DI, HL, and urinary symptoms are common presentations (2, 16, 18, 19). All patients were diagnosed as central DI after water deprivation tests followed by pituitrin or minirin administration, more common in current cohort. The limited number of cases may account for the difference. However, the delayed diagnosis of DI due to overlapping symptoms with DM should be avoided clinically.

Diabetes mellitus was believed to occur in the first decade of life, OA during the early second decade, DI and HL during the second decade, and urological and neurological abnormalities during 10–30 years (2, 16, 18, 20, 21). In our cohort, DM was almost developed in the early first decade, IGT developed in one’s 19 years. OA, DI, and HL were developed during the first and second decades. UD was developed during 10–15 years. DM or IGT was present as the first or second clinical features. There were various combinations of different clinical features and onset age, suggestive of the importance of comprehensive clinical evaluation.

In our cohort, DM was early onset and insulin-dependent. There was a lack of ketosis and autoimmune antibody to glutamic acid decarboxylase and islet cell, distinguished from type 1 DM. These young people with diabetes should be alert to hypoglycemia. Incidences of diabetic retinopathy and nephropathy were both 20% during the first 15 years of DM course, and present as nonproliferative retinopathy and moderately increased albuminuria, respectively, suggesting a low prevalence of diabetic microangiopathic complications. These features were also described in a previous study (22).

Bilateral, progressive OA and high-frequency sensorineural HL urological abnormalities were described like previous publications (2, 20, 22). Low-frequency sensorineural HL may also occur due to the progression of disease or aging. Neurological disorder was found only in one patient, and there was no decease due to the younger age of our cohort.

Genetic analysis of WS patients is given more attention to by physicians. Compound heterozygotic mutations in *WFS1* were identified in Case 3, a combination of family pedigree, supporting the inheritance mode of autosome recessive. The deletion mutation in exon 8 was described as pathogenic before (23), while the frameshift mutation in exon 4 caused by the deletion of 21 bases was considered as a novel pathogenic mutation. No mutation of *WFS1* was identified in Case 1, with a clear family history, and this may suggest undetectable mutation in *WFS1* by regular Sanger sequencing or even new causative gene related to WS may exist. Further basal research may reveal underlying molecular mechanism.

Our experience clearly indicates that WS is a rare, degenerative, and complicate syndrome, characterized by DIDMOAD phenotype. Early-onset DM is insulin-dependent and non-autoimmune, with a low prevalence of complications. Urinary abnormalities may cause renal failure in young patients, and it is advised for urine catheterization and cystostomy at the appropriate time. Genetic analysis should be considered as an effective method to assist diagnosis and genetic consultation. We recognize the limitations of a retrospective study design and limited case numbers. However, our series of patients is the first large series from China which describes the clinical features of WS. The novel mutation in *WFS1* enriches its gene mutation spectrum.

## Conclusion

Wolfram syndrome is a rare, degenerative, and hereditary disease, characterized by early-onset non-autoimmune DM and bilateral progressive OA. It is important to distinguish DM in WS from type 1 DM early. The phenotype is complicated and variable, so it is advised for a comprehensive evaluation of clinical features and a regular follow-up. It is important to carry out urine catheterization and cystostomy for patients with severe urinary abnormalities. Genetic analysis is recommended when suspected of WS. There are no effective measures to prevent disease from progression, and treatments based on symptoms may improve life quality and prolong life expectancy.

## Ethics Statement

This study was carried out in accordance with the recommendations of clinical study guidelines, the Institutional Review Board and Ethics Committee of Peking Union Medical College Hospital (PUMCH), with written informed consent from all subjects. All subjects gave written informed consent in accordance with the Declaration of Helsinki. The protocol was approved by the Institutional Review Board and Ethics Committee of Peking Union Medical College Hospital (PUMCH).

## Author Contributions

FG is the corresponding author: substantial contributions to the conception or design of the work; final approval of the version to be published. LD and QL are equal contributors: substantial contributions to the conception or design of the work; the acquisition, analysis, interpretation of data for the work; drafting the work; revising it critically for important intellectual content. A-LT, J-FM, MY, TY, and X-FC are contributors: direct doctor of some patients in this paper; agreement to be accountable for all aspects of the work in ensuring that questions related to the accuracy or integrity of any part of the work are appropriately investigated and resolved.

## Conflict of Interest Statement

The authors declare that the research was conducted in the absence of any commercial or financial relationships that could be construed as a potential conflict of interest.
