# Supplementary material for: Clinical Characteristics of Wolfram Syndrome in Chinese Population and a Novel Frameshift Mutation in WFS1
Source: Front Endocrinol (Lausanne). 2018 Feb 12;9:18. doi: 10.3389/fendo.2018.00018 (PMC5816339; doi:10.3389/fendo.2018.00018)
Supplement: Supplementary file 1 [file table_1.doc]

**Supplementary Table 1∣The onset ages of different clinical features in Patients with WS**

| **Case No.** | **DM/IGT** | **OA** | **DI** | **HL** | **UD** |
| --- | --- | --- | --- | --- | --- |
| 1 | 5 | 6 | 2 | 5 | No |
| 2 | 4 | 15 | 14 | 5 | 15 |
| 3 | 3 | 5 | 9 | 9 | 10 |
| 4 | 19 | 19 | 15 | No | No |
| 5 | 1 | 10 | 13 | No | 14 |
| 6 | 4 | 13 | 11 | 14 | 14 |
| min | 1 | 5 | 2 | 5 | 10 |
| median | 4 | 11.5 | 12 | 7.7 | 14 |
| max | 19 | 19 | 15 | 14 | 15 |
| mean | 6 | 11.3 | 10.7 | 8.3 | 13.3 |
| SD | 2.7 | 2.2 | 1.9 | 2.1 | 1.1 |

Abbreviations: DM, diabetes mellitus; IGT, impaired glucose tolerance; OA, optic atrophy; DI, diabetes insipidus; HL, hearing loss; UD, urological disorder.
