# Supplementary material for: Clinical Characteristics of Wolfram Syndrome in Chinese Population and a Novel Frameshift Mutation in WFS1
Source: Front Endocrinol (Lausanne). 2018 Feb 12;9:18. doi: 10.3389/fendo.2018.00018 (PMC5816339; doi:10.3389/fendo.2018.00018)
Supplement: Supplementary file 2 [file table_2.doc]

**Supplementary Table 2∣Results of genetic analysis and family history in Patients with WS**

| **Case No.** | **Family history details** | **Genetic analysis** |
| --- | --- | --- |
| 1 | Father had hearing loss and IGT; mother had optic nerve atrophy and IGT; older sister had DM, urinary abnormalities, and optic nerve atrophy, died of renal failure in 9 years | No mutation detected in  *WFS1,* except for 3 SNP (details unknown) |
| 2 | Grandfather had DM | 3 pathogenic mutations  detected in exon 8 in *WFS1* |
| 3 | Father had cogenital poor eyesight; mother had hearing loss | 2 pathogenic mutations (details unknown)  detected in exon 8 and exon 4 respectively in *WFS1* (details shown in Figure 2) |
| 4 | None | 3 pathogenic mutations  detected in exon 8 and  5 SNP in *WFS1*  (details unknown) |
| 5 | Mother had DM | Had not been analyzed |
| 6 | None | Had not been analyzed |

Abbreviations: DM, diabetes mellitus; IGT, impaired glucose tolerance; SNP, single ncleotide polymorphism.
